# Compact Hybrid Multi-Color Space Descriptor Using Clustering-Based Feature Selection for Texture Classification

**DOI:** 10.3390/jimaging8080217

**Published:** 2022-08-08

**Authors:** Mohamed Alimoussa, Alice Porebski, Nicolas Vandenbroucke, Sanaa El Fkihi, Rachid Oulad Haj Thami

**Affiliations:** 1 UR 4491, LISIC, Laboratoire d’Informatique Signal et Image de la Côte d’Opale, Univ. Littoral Côte d’Opale, F-62100 Calais, France; 2 Information Retrieval and Data Analytics Group, ADMIR Laboratory, Rabat IT Center, ENSIAS, Mohammed V University in Rabat, Rabat BP 713, Morocco

**Keywords:** color texture representation, texture classification, color spaces, dimensionality reduction, feature selection, color local binary pattern histogram, chromatic cooccurrence matrix

## Abstract

Color texture classification aims to recognize patterns by the analysis of their colors and their textures. This process requires using descriptors to represent and discriminate the different texture classes. In most traditional approaches, these descriptors are used with a predefined setting of their parameters and computed from images coded in a chosen color space. The prior choice of a color space, a descriptor and its setting suited to a given application is a crucial but difficult problem that strongly impacts the classification results. To overcome this problem, this paper proposes a color texture representation that simultaneously takes into account the properties of several settings from different descriptors computed from images coded in multiple color spaces. Since the number of color texture features generated from this representation is high, a dimensionality reduction scheme by clustering-based sequential feature selection is applied to provide a compact hybrid multi-color space (CHMCS) descriptor. The experimental results carried out on five benchmark color texture databases with five color spaces and manifold settings of two texture descriptors show that combining different configurations always improves the accuracy compared to a predetermined configuration. On average, the CHMCS representation achieves 94.16% accuracy and outperforms deep learning networks and handcrafted color texture descriptors by over 5%, especially when the dataset is small.

## 1. Introduction

As texture and color are two salient visual cues in human perception, color textures provide essential information for object recognition and scene understanding. Therefore, color texture analysis is widely used in many computer vision applications. The development of several benchmark color image databases shows the interest of the scientific community in addressing imaging applications by color texture classification [1,2,3,4]. Color texture classification is the process of predicting the class of input data among a set of categories by the analysis of their colors and their textures. In traditional approaches, this process requires defining a descriptor to represent and discriminate the different texture classes, taking their inter-class and intra-class appearance variations into account. Thus, texture classification is typically categorized into two sub-problems of representation and decision [5]. Texture representation is the step that consists of extracting features that describe color texture information where both the spatial organization of pixels in the image plane and the distribution of their colors in a color space are considered. Numerous descriptors have been proposed in recent decades to represent color textures [6]. These representations can be divided into two categories depending on the descriptors are handcrafted (theory-driven representation) or designed directly from the data (data-driven representation). This paper focuses on the former with a comparison to the latter.

To address a color texture classification problem with handcrafted descriptors, it is first necessary to choose a color space and a texture descriptor which are well suited to the application. Most of these descriptors use parameters that must be carefully tuned depending on the application. The representation of the color textures then consists of extracting color texture features from the chosen descriptor which is computed from images coded in the chosen color space. These choices directly impact the classification performance of color textures. To overcome the problem of a prior choice, many authors propose to combine either multiple descriptors [7,8] and/or multiple color spaces [9,10,11], or manifold parameter settings of a same descriptor [12,13,14] but no work simultaneously combines these three elements for color texture classification purposes. The choice or the combination of different texture descriptors and color spaces, as well as the suitable adjustment of their parameters to produce interpretable, flexible, robust, invariant, and compact descriptors for color texture classification are still open problems. To address this issue, this paper proposes an original approach that simultaneously takes into account the properties of several configurations of different descriptors computed from images coded in multiple color spaces. In order to highlight the contribution of the proposed approach which combines together three key elements of color texture representation (color spaces, texture descriptors and parameter settings), comparisons are performed when only two, one or none of these elements are combined. For fair assessments, standard descriptors and a basic classifier are used.

Since the proposed approach generates a high-dimensional representation with irrelevant or redundant color texture features, it suffers from the curse of dimensionality that appears especially when the number of features is too large compared to the number of training samples [15]. This phenomenon requires a dimensionality reduction scheme to improve the performance of the used classifier in terms of accuracy and computation time. Such a scheme can be achieved either by feature extraction or by feature selection during a learning process. Feature extraction techniques reduce the feature space dimensionality by transforming the original feature space into a new reduced-size feature space. However, this transformation leads to lose the units and the explainability of the original feature space. Moreover, such a transformation requires the computation of the initial feature set to obtain the new reduced feature space, which could be time consuming. That is the reason why feature selection is here preferred. The goal of feature selection is to find a relevant subset from the initial feature space that can improve the overall performance of a classification algorithm with a better understanding of the data [16,17]. When dealing with high-dimensional data, many feature selection approaches can successfully remove irrelevant features but fail to pull redundant ones out [18,19]. To overcome this problem, several feature selection algorithms that use feature clustering were proposed in recent decades in both supervised and unsupervised contexts [18,20,21,22,23,24]. This paper focuses on clustering-based feature selection approaches in a supervised context. These approaches aim to divide the initial feature space into a set of groups called clusters so that the features of a same cluster are considered to be redundant. This leads to the selection of one feature to represent each cluster. The resulting feature subset is thus considered to be relevant and non-redundant [20]. Clustering-based feature selection algorithms can outperform the traditional feature selection methods by reducing the redundancy, reaching high accuracy and, in some cases, reducing the calculation time. Even though they have recently gained much attention, their number is still relatively limited and they need parameters to be adjusted [18]. In this context, we have previously proposed a clustering-based sequential feature selection approach for the classification of high-dimensional data where the feature clustering stage is fully automatic and does not require any parameter adjustment [9]. In this paper, we consider an adapted version of this approach which follows three stages. First, an automatic feature clustering algorithm is applied in order to divide the feature set into a number of clusters in which features are correlated. Then, one feature is sequentially selected per group to construct feature spaces with different dimensions. Finally, the dimensionality of the final feature space is determined by using the accuracy of a classifier.

**Figure 1:**
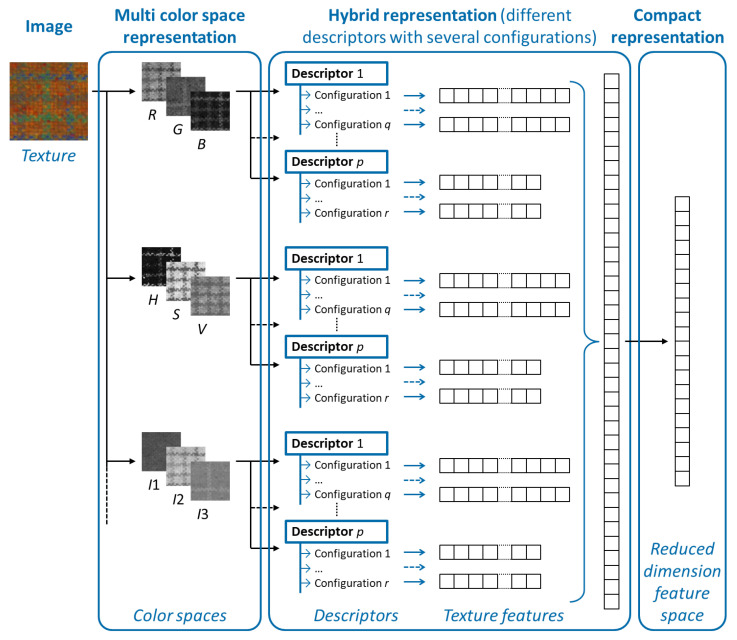
The representation of a texture by the proposed compact hybrid multi-color space descriptor: the image of a color texture is firstly coded in multiple color spaces. Then, several configurations of *p* descriptors are used to generate a large set of color textures features. Finally, a dimensionality reduction scheme is applied to represent the color texture.

Based on this scheme, this paper proposes a compact, hybrid and multi-color space (CHMCS) representation where texture features are computed and selected from several configurations of different descriptors and with multiple color spaces in order to simultaneously take into account various spatial and color properties. By combining the features, the proposed approach thus overcomes the difficulty of a prior choice of a relevant descriptor, a well suited configuration of each descriptor and an appropriate color space. It aims thus to provide a comprehensible and interpretable representation of textures. Figure 1 illustrates how a texture is represented by the CHMCS descriptor. This figure shows the different stages of the proposed representation which are detailed in the next sections of this paper.

After having briefly presented related work on color texture representation in Section 2, Section 3 details how color texture features are extracted from several configurations of two different descriptors computed with images coded in multiple color spaces. Since the representation of the color textures with this feature set generates a high-dimensional feature space, Section 4 presents how a compact hybrid multi-color space (CHMCS) descriptor is derived from this space. This descriptor is based on a dimensionality reduction scheme that uses a clustering method for selecting the most discriminating features with acceptable processing times. In Section 5, experiments are carried out on five benchmark color texture databases. The experimental results show the relevance of the CHMCS descriptor and highlight its performances compared to traditional color texture descriptors and deep learning approaches.

## 2. Related Work

### 2.1. Color Texture Representation

A very large number of texture descriptors have been proposed in recent decades [5]. Most of them have been developed or extended from their gray level definition in order to represent color textures [6]. With the emergence of deep learning, color texture descriptors have evolved from theory-driven descriptors which provide color texture features based on manually defined models into data-driven descriptors which are directly designed from image data.

Most data-driven approaches use convolutional neural networks (CNNs) or pretrained CNNs where a large number of free parameters are determined by training. Popular CNN architectures such as AlexNet, GoogleNet, ResNet or visual geometry group (VGG) have been proposed in the last decade [25,26,27,28]. They are trained on very large datasets and can be easily transferred to many other problems including texture classification. Liu et al. divided CNN-based texture representation methods into three categories: (1) using pretrained generic CNN models where the network is used as a feature extractor that generates features then used by a standard classifier; (2) using fine-tuned CNN models where a training dataset of a specific texture classification task is submitted to the pretrained network in order to fine-tune it (end-to-end learning); and (3) using handcrafted deep CNN where some parameters of the network are predetermined [5]. Several studies have shown that CNN-based methods are suitable for color texture classification tasks [7,13]. Although these deep learning and transfer models provide impressive and, on average, superior performances compared to other approaches, the generated representations and the decisions taken can be difficult to understand. In addition, they suffer from their dependence on training data and tend to overfit with small training sets. To overcome this problem, different image data augmentation approaches have been proposed [29]. Moreover, the performance of CNN-based methods seems to decrease when dealing with fine-grained images, such as color textures [13]. Conversely, the results reached with the theory-driven approaches can be more easily explained and interpreted. Theory-driven descriptors are able to address applications that require fine-grained color texture representations and/or where only limited amounts of labeled training data may be available.

The traditional handcrafted descriptors combine spatial and color information following two main approaches depending on whether they are considered jointly or independently to generate color texture features [30,31]. In these approaches, color textures can be represented using different color spaces [10,32,33]. When spatial and color information are jointly considered, the color texture features take into account the spatial relationships within each of the three color components of the used color space (marginal representation). In addition, the spatial interactions between the color components of neighbor pixels can also be exploited by considering the opposing pairs of color components (opponent component representation). For example, Palm proposed the integrative cooccurrence matrices that combine single-channel cooccurrence matrices applied to a separate color component (for example, the *R*, *G* and *B* channels of a color image) and multi-channel cooccurrence matrices which capture the correlation between textures of different color channels (for example, the *R*-*G*, *R*-*B* and *G*-*B* pairs of channels of a color image) [34]. This concept was extended to the reduced size chromatic cooccurrence matrix (RSCCM) where the quantization level of the color component is decreased to compute cooccurrence matrices within and between color components from which inter-channel and intra-channel features can be extracted [35]. This strategy was also applied to the well-known local binary pattern (LBP) operator in which neighbor pixels are thresholded with the value of the central pixel to give a binary number that codes the local pattern. Several LBP variants have thus emerged: opponent color local binary patterns (OCLBP) where LBP is computed on each color channel separately and from pairs of channels [30]; extended color local binary patterns (EOCLBP), where symmetric pairs of color channels are additionally taken into account [36]; improved opponent color local binary patterns (IOCLBP) in which thresholding is performed with the average value of the neighbor pixels [37]; local color vector binary patterns (LCVBP) that concatenate color norm patterns (thresholding is achieved with the color norm of the pixels that jointly take the three color channels into account); and color angular patterns (the ratio of pixel values between pairs of color channels is used for thresholding) [38], spatially weighted order binary pattern (SWOBP) where a multi-channel color order pattern is used to jointly encode inter-channel features [39].

Choosing the right descriptor and color space for classifying textures is obviously a crucial but difficult problem to solve, agreeing that classification results depend on the choice of the color texture features as well as the tuning of their parameters. To overcome this problem, many authors have proposed to combine various texture descriptors and/or several color spaces in order to take into account their different properties [7,8,9,10,11,40,41]. Porebski et al. proposed a multi color space approach that selects EOCLBP histograms computed from images coded in nine different color spaces [10]. Considering spatial and color information independently, Khan et al. proposed to combine five texture descriptors with a pure color descriptor to obtain a single heterogeneous color texture representation [8]. Cusano et al. showed that the use of an ensemble of twelve handcrafted image descriptors in a multiple classifier framework increased the classification accuracy of texture images acquired under different lighting conditions [7]. They also combined this representation with convolutional neural networks to improve the classification performance. Banerji et al. proposed a 3D-LBP descriptor that they combined with the histograms of oriented gradients of its wavelet transform to produce a novel descriptor [11]. They showed that the fusion of color texture features extracted from this descriptor with seven different color spaces achieves a significantly better image classification performance than with each color space individually considered. Recently, Alimoussa et al. proposed extracting color texture features from two different descriptors and five color spaces which are combined in a single larger set of features [9].

All these works show the relevance of combining different descriptors and/or color spaces to classify textures. In most of them, the configurations of the used descriptors are predefined beforehand with a standard setting of their parameters. However, the texture properties of the different classes may be so variable that they require being represented with different descriptor configurations. Moreover, the appearance of textures can vary due to the change of observation conditions (illumination, field of view, spatial resolution, orientation, viewpoint, deformation, etc.) and texture representation has to take into account possible intra-class property variations. Pioneer work that combines the different configurations of a same descriptor for color texture classification issues has been proposed by Mäenpää et al. and performed with the multi-resolution LBP operator by concatenating the histograms produced by this descriptor with different parameters [30]. This multi-scale strategy was then applied by other authors and extended to different variants of the LBP descriptor [12,42]. Bello-Cerezo et al. led a comparative study between off-the-shelf CNN-based features and handcrafted image descriptors from which multiple resolution feature vectors are extracted from each descriptor [13]. This multi-resolution representation is individually applied to different versions of the LBP operator, cooccurrence matrices, histograms of oriented gradients, image patch-based classifier and Gabor features in order to evaluate the performance of color texture classification obtained with each image descriptor and each compared deep learning network. Promising results are also provided by Alimoussa et al. that combine various configurations of EOCLBP and RSCCM descriptors independently considered [14]. Although all these works show the interest of considering several parameter settings of a same descriptor, they do not simultaneously take into account several descriptors with different configurations. The synthesis of these works shows that the authors have combined either multiple descriptors and/or multiple color spaces or several parameter settings of a same descriptor but, to our knowledge, no work has attempted to simultaneously combine these three elements for color texture purposes. The main contribution of this paper is to address this issue that requires a dimensionality reduction because of the high number of generated candidate color texture features.

### 2.2. Dimensionality Reduction by Feature Selection

Feature selection is an essential technique to reduce the dimensionality of a feature space for data classification purposes. It contributes to improve the performances in terms of prediction quality, computation time and model understanding. From a set of available features, it aims to generate a low-dimensional feature subset without irrelevant or redundant features. Feature selection has become the focus of many research applications especially when datasets tend to be huge. However, traditional feature selection methods are not suitable for large dimensional feature spaces [43]. This is the reason why approaches that use feature clustering techniques have more recently gained attention for their ability to improve the selection process.

Classical feature selection methods can be achieved by two main models named “filter” and “wrapper” [44]. Filter models deploy statistical measures to evaluate the discriminating power of features or subsets of features, whereas wrapper models compute the accuracy reached with a particular classifier to guide the search for determining the most discriminating feature subset. Other techniques, called “hybrid” or “embedded” models, combine both filter and wrapper approaches [16]. On the one hand, wrapper models tend to achieve better results than filter ones but suffer from a high computational cost since they depend on a classifier [19,45]. On the other hand, filter models are simple to design, classifier independent and faster. The embedded models that we propose to use in this paper take advantage of the speed of a filter model as well as the selection quality of a wrapper one.

In a supervised learning context, a feature selection method can be performed on a training dataset either by feature ranking or by feature subset search. Feature ranking algorithms individually rank features in order to select the most discriminating ones. Therefore, they are fast and easy to apply. However, it has been shown that the combination of individually relevant features does not necessarily yield a high classification performance [46]. This is mainly due to the non consideration of the interactions and the redundancy that may exist between features. To overcome this problem, a feature subset search, which evaluates groups of features, is preferred.

In the same way, clustering-based feature selection approaches can be performed either by feature ranking or by feature subset search. In each case, a filter or a wrapper model can be associated, leading to different strategies introduced in the following sections [9].

#### 2.2.1. Clustering-Based Feature Ranking Approaches

In clustering-based feature ranking approaches, the feature space is firstly divided into a number of groups by means of a clustering algorithm. The proposed approaches differ depending on the clustering algorithm used. Then, the clusters and/or the features in each cluster are ranked in order to select the representative features of each group. The feature selection is either performed with the filter model [18,47] or with the wrapper model [22]. Harris and Niekerk proposed a feature clustering and ranking (FCR) approach where feature clustering is performed using the affinity propagation algorithm associated with a correlation coefficient as a similarity measure [21]. A single feature from each of the top ranked clusters is then selected by using either a filter model or a wrapper one according to two different evaluation measures.

#### 2.2.2. Clustering-Based Feature Subset Search Approaches

Most of the clustering-based feature subset search approaches associate a clustering algorithm with a wrapper model to evaluate the feature subset relevance following three main schemes. In the first one, clustering is applied as a pre-processing stage where only one feature is selected from each group to constitute the feature set from which a feature subset search is performed [48]. Other schemes cluster the initial feature set into a predefined number of groups, and then evaluate the relevance of each group in order to remove irrelevant feature groups before merging the remaining groups and repeating the whole scheme [24]. In the last kind of scheme, the feature subset search is applied in each group defined by the clustering algorithm and the features selected from each group are merged to form the final selected feature subset [23].

Apart from the one we previously proposed, very few approaches use a filter model with a clustering-based feature subset search [9]. We showed that this approach provides a high level of dimensionality reduction, high classification accuracy with a reasonable processing time and a limited number of parameters to be adjusted compared to other feature selection methods presented here. The selection approach proposed in this paper is inspired from this clustering-based filter feature selection method. It takes advantage of its fast filter model for searching feature subsets with different dimensions and adds a wrapper model to determine the dimensionality of the relevant feature space, thereby building a clustering-based embedded feature selection approach described in Section 4.

## 3. Color Texture Features

In this paper, we propose to apply our approach with two popular texture descriptors known for their computational simplicity: the reduced size chromatic cooccurrence matrix (RSCCM) [34,35] and the extended opponent color local binary pattern (EOCLBP) [10,36]. These two descriptors are computed from images coded in different color spaces and require defining a neighborhood N in which the spatial interactions within and between the color components of neighbor pixels are both taken into account. For the first descriptor, Haralick features are extracted from different configurations of RSCCM. For the second one, we propose extracting statistical features from the histograms of many EOCLBP configurations [14].

### 3.1. Color Spaces

Color images are usually acquired by devices that code the colors in the RGB color space. However, the color of pixels can be represented in different color spaces which respect different physical, physiologic, and psycho-visual properties. These color spaces can be categorized into four families: the primary spaces; the luminance–chrominance spaces; the perceptual spaces; and the independent color component spaces [35].

Since the choice of a color space directly impacts the classification results, many authors have tried to compare the results obtained using different color spaces in order to find the most suited one for a given application [30,32,33]. The synthesis of these works shows that there is no color space well suited to represent all types of textures. To solve this problem, a few studies have proposed multi-color space approaches [10]. These approaches simultaneously exploit the properties of multiple color spaces by combining them and thus overcome the difficulty of choosing a single relevant color space. Although these approaches have shown their relevance with variable numbers of considered color spaces, it appears that a limited number of color spaces representative of each family is sufficient to improve classification performances [3,10]. Moreover, many of these spaces require knowing the properties of the illumination and the acquisition system to be independent of the device. As these parameters are not known for all image datasets, we propose to describe textures with only device-dependent color spaces that do not need this knowledge. In addition to the RGB color space, a color space belonging to each of the four families is considered herein:

- $R_{n}G_{n}B_{n}$ normalized primary space obtained by dividing each color component value by the sum of the three ones;
- YCbCr luminance–chrominance space which separates the achromatic and chromatic signals for the television signal transmission;
- I1I2I3 independent color component space which provides the less correlated components as possible;
- HSV perceptual space which attempts to quantify the subjective human color perception using the intensity, the hue, and the saturation components with the hexcone model.

**Figure 2:**
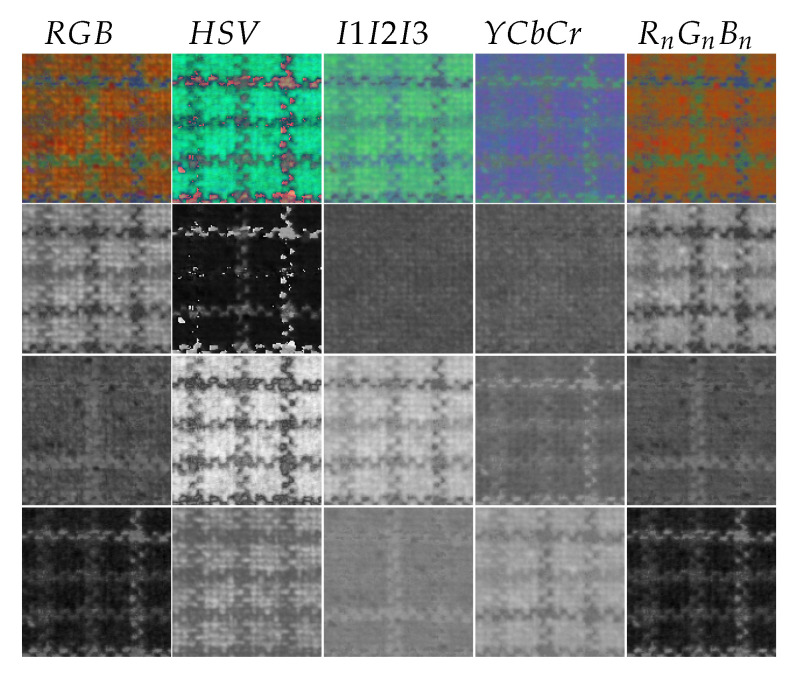
Image of a texture acquired in the RGB color space and converted into the HSV, I1I2I3, YCbCr and $R_{n}G_{n}B_{n}$ device-dependent color spaces.

Figure 2 shows the image of a texture acquired in the RGB color space and converted into each of the used color spaces. Converted images are displayed in false colors by additive mixing and their three color channels are displayed below in grayscale. This figure highlights that the texture can be perceived with different appearances depending on the color component and shows the interest of combining multiple color spaces.

The subsequent sections describe the color textures features extracted from images coded in these five three-dimensional spaces whose color components are denoted $C_{1}$, $C_{2}$ and $C_{3}$ for a given $C_{1}C_{2}C_{3}$ color space.

### 3.2. Haralick Features Extracted from Chromatic Cooccurrence Matrices

This section presents the chromatic cooccurrence matrix and its possible configurations from which features are extracted to describe a color texture.

#### 3.2.1. Chromatic Cooccurrence Matrices

This descriptor is the extension to color of the gray level cooccurrence matrix (GLCM) operator that is considered as a two-dimensional histogram of gray level pairs of neighbor pixels. An important property of this operator is its invariance to orientation changes when all the directions of neighbor pixels are taken into account. The chromatic cooccurrence matrix (CCM) considers both the spatial interactions within and between the color components of neighbor pixels in the image plane and the color distribution in a color space [34,35].

Let *Q* be the number of levels used to quantify the color components $C_{1}$, $C_{2}$ and $C_{3}$ of a given color space. A reduced size chromatic cooccurrence matrix (RSCCM) is a Q×Q CCM, where the parameter *Q* is reduced in order to decrease the memory storage cost and thus, the time required to extract texture features from these matrices [35].

The normalized RSCCM $m_{N}^{C_{g},C_{g^{\prime }}}\left[I\right]$ measures the spatial interactions in the neighborhood N between the two color components $C_{g}$ and $C_{g^{\prime }}$ ($g,g^{\prime }\in \left\{1,2,3\right\}$) of an image I. In addition to the quantization level *Q*, the neighborhood N is a second parameter defined by the user.

For an image coded in a color space $C_{1}C_{2}C_{3}$ with a quantization level *Q* and a given neighborhood N, six normalized RSCCMs can be computed:

- Three within-component matrices ($g=g^{\prime }$) $m_{N}^{C_{1},C_{1}}\left[I\right]$, $m_{N}^{C_{2},C_{2}}\left[I\right]$ and $m_{N}^{C_{3},C_{3}}\left[I\right]$;
- Three between-component matrices ($g\neq g^{\prime }$) $m_{N}^{C_{1},C_{2}}\left[I\right]$, $m_{N}^{C_{1},C_{3}}\left[I\right]$ and $m_{N}^{C_{2},C_{3}}\left[I\right]$ where $m_{N}^{C_{g},C_{g^{\prime }}}\left[I\right]$ and $m_{N}^{C_{g^{\prime }},C_{g}}\left[I\right]$ are symmetric.

The choice of component pairs (within and between component matrices) can be viewed as a third parameter of this descriptor. The next subsection deals with the different configurations that an RSCCM can take.

#### 3.2.2. RSCCM Configurations

Before calculating a chromatic cooccurrence matrix, several parameters have to be set and adjusted. This configuration is complex when the color and spatial properties of the analyzed textures are heterogeneous. This principally depends on:

- The number of normalized RSCCM taken into account for a given color space;
- *Q*, the quantization level that defines the size of the RSCCM;
- N, the pixel neighborhood in which cooccurrences are counted. N is controlled by two other parameters:
  - The neighborhood direction: four two-directional neighborhoods are usually used to compute direction-dependent cooccurrence matrices: $0^{{}^{\circ}}$, $45^{{}^{\circ}}$, $90^{{}^{\circ}}$, and $135^{{}^{\circ}}$. In order to simultaneously take into account all the possible directions of an observed texture, an isotropic 3×3 neighborhood is generally used with a number of eight neighbors located in the four directions.
  - The neighborhood distance: this distance, denoted δ, is the spatial infinity-norm distance separating each pixel from its neighbors.

**Figure 3:**
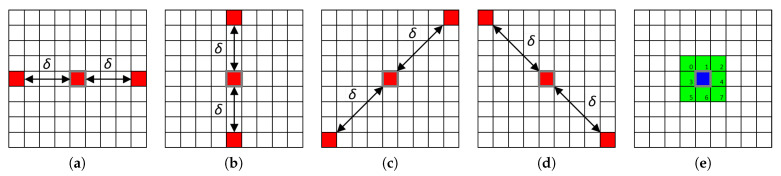
Possible parameter settings of the RSCCM descriptor in the *RGB* color space: (**a**) 2-neighborhood and 0° direction with the $\left(R,R\right)$ component pair; (**b**) 2-neighborhood and $90^{{}^{\circ}}$ direction with the $\left(R,R\right)$ component pair; (**c**) 2-neighborhood and $45^{{}^{\circ}}$ direction with the $\left(R,R\right)$ component pair; (**d**) 2-neighborhood and $135^{{}^{\circ}}$ direction with the $\left(R,R\right)$ component pair; and (**e**) $\left(3\times 3\right)$ 8-neighborhood with the $\left(B,G\right)$ component pair.

Figure 3 gives some examples of RSCCM configurations. Examples (a–d) show configurations using the four directional 2-neighborhoods $0^{{}^{\circ}}$, $90^{{}^{\circ}}$, $45^{{}^{\circ}}$ and $135^{{}^{\circ}}$, respectively, at the distance δ associated with the *R* color component of the RGB color space so that within-component matrices are computed with the $\left(R,R\right)$ component pair. Example (e) shows a configuration using the 3×3 isotropic 8-neighborhood (δ=1) associated with the $\left(B,G\right)$ component pair such that a between-component matrix can be computed.

In this paper, an isotropic $\left(2\times \delta +1\right)\times \left(2\times \delta +1\right)$ neighborhood is used with a number of eight neighbors located in the four directions. Thus, we propose to adjust RSCCM configurations depending on two parameters: the quantization level *Q* and the neighborhood distance δ, assuming that these two parameters control the representation of textures acquired with different observation conditions. Haralick features are thus extracted from RSCCM configurations resulting from each of the following pairs (δ,Q):
(1, 16)(1, 32)(1, 64)(1, 128)(1, 256)(2, 16)(2, 32)(2, 64)(2, 128)(2, 256)(3, 16)(3, 32)(3, 64)(3, 128)(3, 256)(5, 16)(5, 32)(5, 64)(5, 128)(5, 256)(10, 16)(10, 32)(10, 64)(10, 128)(10, 256)

#### 3.2.3. Haralick Features Extracted from RSCCM

The cooccurrence matrices are able to represent the texture but they are not directly used for color texture classification purposes because of the large amount of information they contain. To reduce it while preserving the relevance of these descriptors, Haralick proposed statistical features that can be extracted from each matrix [34]. We propose using the first 13 Haralick features: energy, homogeneity, contrast, correlation, variance, inverse difference moment, sum average, sum entropy, entropy, difference variance, difference entropy, and two measures of correlation I and II [49].

A color texture is then represented by Haralick features extracted from RSCCM with different parameter settings and computed from images coded in multiple color spaces.

### 3.3. Texture Features Extracted from Color Local Binary Pattern Histograms

This section presents the color local binary pattern and its possible configurations from which features are extracted to describe a color texture.

#### 3.3.1. Color Local Binary Pattern Histograms

Color LBPs are extensions to the color of the local binary pattern (LBP) operator that captures the local texture properties of a gray level image [36]. An important property of this operator is its invariance to monotonic gray-scale changes caused, for example, by illumination variations. In order to characterize the whole color texture image, the LBP operator is applied on each pixel and for each pair of components in the color space $C_{1}C_{2}C_{3}$. Considering a pair of components $\left(C_{g},C_{g^{\prime }}\right)$, $\left(g,g^{\prime }\in \left\{1,2,3\right\}\right)$, the color LBP labels a pixel with the component $C_{g}$ by thresholding its neighborhood N in the component $C_{g^{\prime }}$ and by encoding the result as a binary number.

The consideration of the Extended Opponent Color LBP (EOCLBP) operator gives rise to nine LBP images:

- Three within-component LBP images ($g=g^{\prime }$) with the pairs $\left(C_{1},C_{1}\right)$, $\left(C_{2},C_{2}\right)$ and $\left(C_{3},C_{3}\right)$;
- Six between-component ($g\neq g^{\prime }$) with the pairs $\left(C_{1},C_{2}\right)$, $\left(C_{1},C_{3}\right)$, $\left(C_{2},C_{3}\right)$, $\left(C_{2},C_{1}\right)$, $\left(C_{3},C_{1}\right)$ and $\left(C_{3},C_{2}\right)$.

The choice of the considered pairs of color components can be viewed as a parameter of this descriptor. The LBP images are usually not exploited directly and most authors prefer to use LBP histograms where histogram bins are considered as texture features [36]. Another original idea is to extract statistical features such as Haralick features from LBP images [50].

Instead of using the bins of EOCLBP histograms, we propose to extract two different types of statistical features from these histograms in order to provide color texture features consistent with those of other descriptors [14]. In order to characterize the textures acquired with different observation conditions, these features are extracted from several EOCLBP configurations presented in the next subsection.

#### 3.3.2. EOCLBP Configurations

Due to its popularity, many variants of the basic LBP operator, such as the rotation invariant LBP or the uniform LBP that reduces its dimensionality, as well as their few extensions to color, have been proposed the last two decades [36,51].

The definition of the original LBP operator with its 3×3 neighborhood has then been generalized by using a circular neighborhood N so that the EOCLBP parameters are defined by:

- The number of LBP histograms taken into account for a given color space;
- A circular neighborhood N controlled by
  - *P*, the number of neighbor pixels that determines the dimensionality of the LBP histograms. For example, a 3×3 neighborhood with P=8 neighbors gives rise to a $2^{8}=256$-dimensional LBP histogram. For each pair of color components, a color texture is thus described by a $2^{P}$-dimensional histogram;
  - δ, the distance between each pixel and its neighbors. This distance is equal to the radius of the circle around the central pixel. Generally, when a neighbor pixel is not confused with the circle, a bilinear interpolation is used to estimate its location. Here, the neighborhood is pre-sampled.

**Figure 4:**
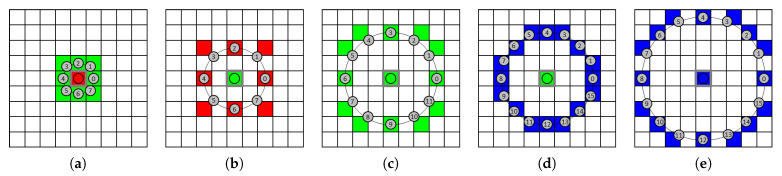
Possible parameter settings of the EOCLBP descriptor in the RGB color space: (**a**) P=8 and δ=1 with the $\left(R,G\right)$ component pair; (**b**) P=8 and δ=2 with the $\left(G,R\right)$ component pair; (**c**) P=12 and δ=3 with the $\left(G,G\right)$ component pair; (**d**) P=16 and δ=3 with the $\left(G,B\right)$ component pair; and (**e**) P=16 and δ=4 with the $\left(B,B\right)$ component pair.

Figure 4 gives some examples of EOCLBP configurations with different values of *P* and δ as well as different pairs of color components in the RGB color space. In these examples, neighbor pixels are numbered from 0 to P−1. The configurations in the examples (a), (b) and (d) are used to compute between-component LBP images while those in examples (c) and (d) allow computing within-component LBP images.

With these parameters, many LBP configurations are available in order to characterize textures in different scales. In this paper, we propose to consider EOCLBP configurations resulting from each of the following pairs (P,δ):
(8, 1)(8, 2)(8, 3)(8, 5)(8, 10)
(12, 2)(12, 3)(12, 5)(12, 10)
(16, 2)(16, 3)(16, 5)(16, 10)

#### 3.3.3. Statistical Features Extracted from EOCLBP Histograms

With the EOCLBP operator, a color texture can be represented by nine LBP histograms that are concatenated to constitute a vector containing $9\times 2^{P}$ features for a given color space $C_{1}C_{2}C_{3}$. Several approaches have been proposed to reduce the dimensionality of such a feature space, such as the uniform LBP operator. Some authors have selected the most discriminant bins that constitute the LBP histograms [36]. Other authors reduced the number of histograms with only three within-component LBP histograms or by adding only three out of six between-component LBP histograms, assuming that the opponent pairs such as $\left(C_{1},C_{2}\right)$ and $\left(C_{2},C_{1}\right)$ are highly redundant [30]. Another approach consists of selecting, among the nine LBP histograms, the most discriminant ones for the considered application [10].

In this paper, we propose to extract statistical features from each LBP histogram to constitute a reduced dimensionality statistical feature vector [14]. For this purpose, two types of statistical features are proposed:

- Six first-order statistical features: mean, median, mode, standard deviation, and two interquartile ranges;
- Eleven second-order statistical features extended from the first 11 Haralick features presented in Section 3.2.3 and adapted to deal with histograms.

## 4. Compact Hybrid Multi-Color Space Descriptor

This section presents the method used to define the proposed CHMCS descriptor which provides a hybrid and compact representation of color textures.

### 4.1. Hybrid Multi-Color Space Representation

By combining the texture features extracted from several configurations of different descriptors computed with images coded in multiple color spaces, a color texture is represented in a high-dimensional feature space.

Let $N_{spac}$, be the number of considered color spaces, $N_{conf}^{p}$, be the number of configurations associated with the pth descriptor and $N_{feat}^{p}$ be the number of color texture features extracted from each configuration of the pth descriptor. The total number *D* of color texture features is given by the relation (1):

(1) $$D=N_{spac}\times \sum_{p}\left(N_{conf}^{p}\times N_{feat}^{p}\right).$$

where $N_{conf}^{p}$ can be computed as the product between $N_{pair}^{p}$, the number of color component pairs considered in each color space for the pth descriptor, and $N_{para}^{p}$, the number of parameter combinations associated with the pth descriptor. Table 1 presents the possible dimensionalities of the feature space depending on these values.

By simultaneously considering the RSCCM and EOCLBP descriptors as well as all the combinations presented in the last two rows of this table, we build a hybrid multi-color space descriptor by concatenating a total number of D=19,695 color texture features. Due to the curse of dimensionality, it is essential to reduce this number in order to define a compact representation of color textures.

### 4.2. Compact Representation

The proposed clustering-based embedded feature selection (CEFS) approach consists of three stages:

1. First, an automatic feature clustering algorithm is applied in order to divide the feature set into a number of clusters in which features are redundant or correlated;
2. Then, one feature is sequentially selected per group;
3. Finally, the dimensionality of the feature space is determined.

The two first stages of the CEFS approach significantly speeds the selection procedure up since a large number of redundant features are eliminated at each step. Indeed, the filter model-based sequential feature selection procedure is applied to all the features belonging to the different clusters so that only one feature per cluster is selected at each iteration step. Features belonging to the same cluster are removed and thus not considered in the next steps of the selection procedure. The feature clustering stage is fully automatic and does not require any parameters to be adjusted. This multi-criterion approach associates two complementary measures presented in the following subsections: a correlation-based criterion to cluster the feature set and a distance-based criterion to evaluate the relevance of each candidate feature space. The last subsection presents the third stage of the CEFS approach which uses the accuracy of a classifier operating in each feature subspace selected at different dimensions in order to determine the relevant feature space dimensionality.

#### 4.2.1. Notations

Let $S=\left\{F_{1},F_{2},\cdots ,F_{k}\cdots ,F_{D}\right\}$ be a set of *D* features where $F_{k}$ is the kth feature of this set. Feature selection aims to define a subset $S_{\hat{d}}\subset S$ with a reduced number $\hat{d}$ of features.

In a supervised context where the class labels of the color texture are known, this procedure is applied on training data of *N* color texture samples. The training data can be represented in the *D*-dimensional feature space by the N×D data matrix X:

$$X=\left(\begin{array}{cccccc} x_{1}^{1} & x_{1}^{2} & \cdots & x_{1}^{k} & \cdots & x_{1}^{D}\\ x_{2}^{1} & x_{2}^{2} & \cdots & x_{2}^{k} & \cdots & x_{2}^{D}\\ \vdots & \vdots & \ddots & \vdots & \ddots & \vdots \\ x_{i}^{1} & x_{i}^{2} & \cdots & x_{i}^{k} & \cdots & x_{i}^{D}\\ \vdots & \vdots & \ddots & \vdots & \ddots & \vdots \\ x_{N}^{1} & x_{N}^{2} & \cdots & x_{N}^{k} & \cdots & x_{N}^{D} \end{array}\right).$$

Each of the *D* columns of the matrix X is the *N*-dimensional *feature* vector $x^{k}={\left(x_{1}^{k},x_{2}^{k},\cdots ,x_{i}^{k},\cdots ,x_{N}^{k}\right)}^{T}\in R^{N}$ that represents a feature $F^{k}$ (k=1,⋯,D) and each of the *N* rows of the matrix *X* is the *D*-dimensional *sample* vector $x_{i}=\left(x_{i}^{1},x_{i}^{2},\cdots ,x_{i}^{k},\cdots ,x_{i}^{D}\right)\in R^{D}$ that represents the ith color texture sample (i=1,⋯,N) so that:

$$X=\left(\begin{array}{c} x_{1}\\ x_{2}\\ \vdots \\ x_{i}\\ \vdots \\ x_{N} \end{array}\right)=\left(\begin{array}{cccccc} x^{1} & x^{2} & \cdots & x^{k} & \cdots & x^{D} \end{array}\right).$$

Let $m=\left(m^{1},m^{2},\cdots ,m^{k},\cdots ,m^{D}\right)$ be the *D*-dimensional mean feature vector where $m^{k}$ is the mean of *N* elements of $x^{k}$ defined by Equation (2):

(2) $$m^{k}=\frac{1}{N}\sum_{i=1}^{N}x_{i}^{k}.$$

The matrix X is associated with an *N*-dimensional vector $y={\left(y_{1},y_{2},\cdots ,y_{i},\cdots ,y_{N}\right)}^{T}\in R^{N}$ that represents the class labels of the training data where $y_{i}$ is the class label of the ith color texture sample (i=1,⋯,N). Let $N_{C}$ be the number of classes.

Let $m_{j}=\left(m_{j}^{1},m_{j}^{2},\cdots ,m_{j}^{k},\cdots ,m_{j}^{D}\right)$ be the *D*-dimensional mean feature vector of class *j* where $m_{j}^{k}$ is the mean of the feature $F_{k}$ computed on the $N/N_{C}$ samples labeled to the jth class:

(3) $$m_{j}^{k}=\frac{N_{C}}{N}\sum_{\underset{y_{i}=j}{i=1}}^{N}x_{i}^{k}.$$

#### 4.2.2. Feature Clustering

The clustering of the feature set S is based on a dependency graph where the nodes are the considered color texture features which are linked by an edge if they are correlated. Two features are correlated if the absolute value of their Pearson’s linear correlation coefficient is higher than a threshold [52]. The correlation ρ between two color texture features $F_{k}$ and $F_{l}$ represented by their vector $x^{k}$ and $x^{l}$, respectively, is defined by the following equation:

(4) $$\rho \left(x^{k},x^{l}\right)=\frac{\sum_{i=1}^{N}\left(x_{i}^{k}-m^{k}\right)\left(x_{i}^{l}-m^{l}\right)}{\sqrt{\sum_{i=1}^{N}{\left(x_{i}^{k}-m^{k}\right)}^{2}\times \sum_{i=1}^{N}{\left(x_{i}^{l}-m^{l}\right)}^{2}}},$$

If $x^{k}$ and $x^{l}$ are totally correlated, the value of ρ tends to its limits 1 or −1, and if they are completely uncorrelated, ρ is close to zero. Features directly connected are considered to be “dependent” and the features which are indirectly connected via other features are considered to be “long dependent”. The proposed clustering algorithm aims to put into the same feature cluster the dependent and long dependent features. Given a correlation threshold, two features $F_{k}$ and $F_{l}$ belonging to S are considered to be long dependent if $\exists F_{m}\in S$, $F_{k}$ is dependent and so, connected to $F_{m}$ and $F_{l}$ is dependent and so, connected to $F_{m}$ in the dependency graph.

As in many clustering algorithms such as K-means and affinity propagation, the parameter setting, generally adjusted by a user, is crucial because it directly impacts the clustering result. The originality of our approach is that it automatically adjusts the correlation threshold which is the only parameter of the clustering algorithm. This operation is performed by varying the correlation threshold and then evaluating the clustering quality.

The clustering algorithm partitions the feature set S into a number $N_{t}$ of clusters depending on the value *t* of the correlation coefficient threshold ($t=\left\{0.75,0.8,0.85,0.9,0.95\right\}$) so that $S=\left\{C_{1},C_{2},\cdots ,C_{a},\cdots ,C_{N_{t}}\right\}$ where $C_{a}$ is the ath cluster of features ($a=1,\cdots ,N_{t}$).

It is assumed that the more the clusters are well separated and compact, the higher the clustering quality is. The clustering quality evaluation is so performed using a measure of cluster separability and compactness defined by Equation (5):

(5) $$Tr\left(S\right)=\mathrm{trace}(\left({\left(W+B\right)}^{-1}\right)\times B),$$

where trace(A) is the trace of the matrix *A*, B is the between-cluster scatter matrix defined by Equation (6) and W is the within-cluster scatter matrix defined by Equation (7).

(6) $$B=\frac{1}{D}\sum_{a=1}^{N_{t}}\left|C_{a}\right|\times \left(\mu^{a}-\mu \right){\left(\mu^{a}-\mu \right)}^{T},$$

(7) $$W=\frac{1}{D}\sum_{a=1}^{N_{t}}\sum_{\underset{F_{k}\in C_{a}}{k=1}}^{D}\left(x^{k}-\mu^{a}\right){\left(x^{k}-\mu^{a}\right)}^{T}.$$

In these equations, $\left|C_{a}\right|$ is the cardinal of $C_{a}$, $\mu ={\left(\mu_{1},\mu_{2},\cdots ,\mu_{i},\cdots ,\mu_{N}\right)}^{T}$, is the *N*-dimensional mean sample vector with $\mu_{i}$, the mean of the *D* elements of $x_{i}$ defined by Equation (8) and $\mu^{a}={\left(\mu_{1}^{a},\mu_{2}^{a},\cdots ,\mu_{i}^{a},\cdots ,\mu_{N}^{a}\right)}^{T}$ is the *N*-dimensional mean sample vector of the cluster *a* ($a=1,\cdots ,N_{t}$) with $\mu_{i}^{a}$, the mean of the ith sample computed on the features belonging to the ath cluster defined by Equation (9):

(8) $$\mu_{i}=\frac{1}{D}\sum_{k=1}^{D}x_{i}^{k}.$$

(9) $$\mu_{i}^{a}=\frac{1}{\left|C_{a}\right|}\sum_{\underset{F_{k}\in C_{a}}{k=1}}^{D}x_{i}^{k}.$$

The correlation threshold $\hat{t}$ used by the clustering algorithm is the one for which Tr is maximum. Let us note that the higher the correlation threshold is, the less the number of initial connections between features is, the less the number of correlated and also long correlated features is, and therefore the greater the number of clusters is.

#### 4.2.3. Sequential Feature Selection

Then, a sequential forward selection (SFS) approach, based on a filter model, is applied to the feature set $S=\overset{N_{\hat{t}}}{\underset{a=1}{⋃}}\left\{C_{a}\right\}$ previously clustered.

Since features in the same cluster are considered redundant, only one feature from each cluster is selected by using the filter model.

Following a forward sequential strategy, the feature selection algorithm selects, at each iteration step, a feature from the candidate feature set depending on the value of the evaluation function. For this purpose, each of the remaining candidate features is added to the feature subset under construction in order to consider as many feature subsets as there are candidate features. As for the feature clustering step, we used a distance-based measure as an evaluation function. Previously, the trace criterion was applied to measure the cluster separability and compactness. In this stage of the feature selection process, this criterion, defined by Equation (5), was used to measure the class separability and compactness and evaluate the discriminating power of a candidate feature space. Here, B represents the between-class scatter matrix defined by Equation (10) where the number of samples $\frac{N}{N_{C}}$ is equal for each class and W represents the within-class scatter matrix defined by Equation (11).

(10) $$B=\frac{1}{N_{C}}\sum_{j=1}^{N_{C}}\left(m_{j}-m\right){\left(m_{j}-m\right)}^{T},$$

(11) $$W=\frac{1}{N}\sum_{j=1}^{N_{C}}\sum_{\underset{y_{i}=j}{i=1}}^{N}\left(x_{i}-m_{j}\right){\left(x_{i}-m_{j}\right)}^{T},$$

where $y_{i}=j$ means that the sum is applied to all the samples whose class label $y_{i}$ is equal to *j*, the index of the considered class.

The selected feature subset at each iteration step *d* of the procedure is the subset for which the trace criterion is the maximum.

Once a feature is added to this subset, the cluster in which this feature belongs is removed to update the remaining candidate feature set that will be evaluated at the next iteration step (d+1). As a consequence, the number of candidate features dramatically decreases at each step. On the one hand, this feature cluster removal reduces the feature redundancy and, on the other hand, this stage accelerates the selection procedure compared to a classical sequential feature selection method.

#### 4.2.4. Determination of the Relevant Feature Space Dimensionality

Finally, the last step of the feature selection scheme consists of determining the dimension of the relevant feature subspace. Since the evaluation function associated with the filter model is monotonic, it cannot be directly used to determine the dimension of the final feature space. The proposed embedded method integrates a classifier whose accuracy is measured once a feature is added at each step *d* of the procedure. For this purpose, the training set is divided into a training image subset and a validation image subset from which the classification accuracy is measured following a *K*-fold evaluation. (K−1) folds are used to constitute a training image subset and the remaining fold is assigned to a validation image subset from which the classification accuracy is measured. This cross-validation procedure is repeated 10 times with different distributions of the training set images in the validation and training subsets [53]. For each *d*-dimensional feature subspace selected at each step *d*, this accuracy is estimated as the mean rate ${\bar{R}}_{d}$ of well-classified validation images over the *K* folds and over the 10 repetitions. Since this measure tends to be stabilized after a limited number of iteration steps, the procedure stops when a maximum number $d_{max}$ of iteration steps is reached or when all the clusters of features are removed. $d_{max}$ is a parameter that controls the learning processing time. The dimension $\hat{d}$ of the selected feature subspace is the one for which ${\bar{R}}_{d}$ is maximum ($d=1,\cdots ,d_{max}$). The accuracy is measured using the nearest neighbor classifier associated with the L1 distance because no parameters need to be adjusted.

#### 4.2.5. Algorithm

Algorithm 1 presents how the CEFS procedure runs.
**Algorithm 1** The CEFS procedure.1.Cluster the feature set S so that $S=\overset{N_{\hat{t}}}{\underset{a=1}{⋃}}\left\{C_{a}\right\}$ (see Section 4.2.2);2.Start with the empty set (d=0, $S_{d}=\emptyset$);3.Add the feature, denoted $F^{*}$, which maximizes $Tr\left(S_{d}\cup \left\{F_{k}\right\}\right)\;|\;F_{k}\in \left\{S\right\}$:
(12)$$F^{*}=\underset{F_{k}\in \left\{S\right\}}{\mathrm{argmax}}\;Tr\left(S_{d}\cup \left\{F_{k}\right\}\right);$$4.Update ($S_{d+1}=S_{d}\cup \left\{F^{*}\right\}$, d=d+1);5.Remove the cluster in which $F^{*}$ belongs ($S=S\setminus C_{a}\;|\;F^{*}\in \left\{C_{a}\right\}$);6.Measure accuracy as the mean rate ${\bar{R}}_{d}$ of well classified validation images;7.Go to 3 if $d\leq d_{max}$ or S=∅;8.End with the computation of the dimension $\hat{d}$, otherwise:
(13)$$\hat{d}=\underset{1\leq d\leq d_{max}}{\mathrm{argmax}}{\bar{R}}_{d}.$$

## 5. Experimental Results

This section firstly presents the image databases on which the experiments were carried out. A first fine analysis of the results reached by our approach on one of these databases is performed. The results obtained with the five databases are then presented, compared, interpreted and discussed.

### 5.1. Datasets

**Figure 5:**
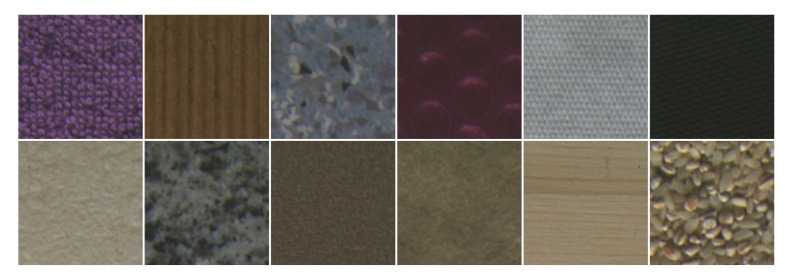
Samples from the Outex dataset among the 68 color texture classes. Each of the 12 surface categories (from top-left to bottom-right: canvas, cardboard, carpet, foam, paper, rubber, tile, granite, sandpaper, wool, wood, and barley-rice) included in this dataset is represented herein by one image.

**Figure 6:**
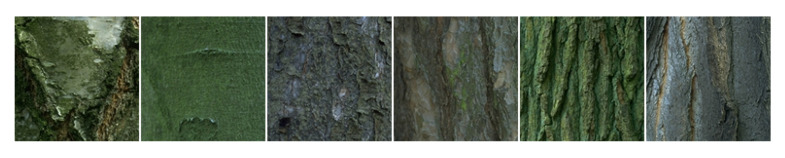
Samples illustrating the six tree bark color textures of the NewBarkTex2 dataset (from left to right: Betula pendula, Fagus silvatica, Picea abies, Pinus silvestris, Quercus robus, and Robinia pseudacacia).

**Figure 7:**
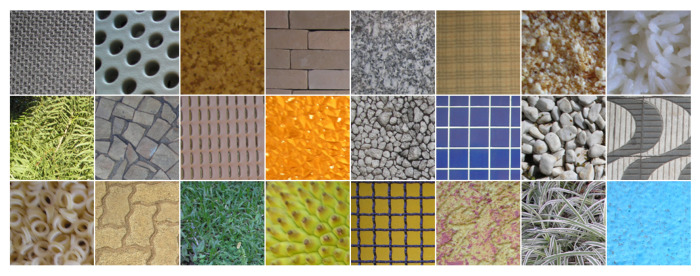
Samples of different categories from the USPtex dataset among the 191 color texture classes.

**Figure 8:**
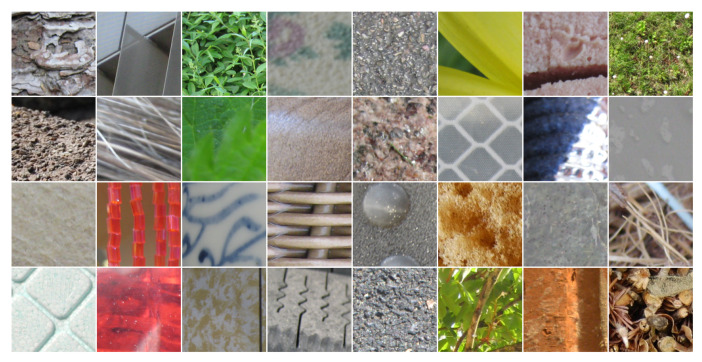
Samples of different categories from the Stex dataset among the 476 color texture classes.

**Figure 9:**
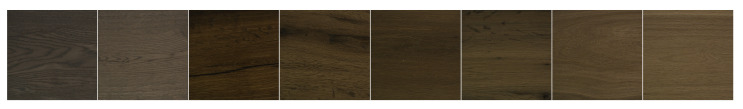
Samples of the OAK wood category from the Parquet dataset among the 38 color texture classes.

In order to highlight the contribution of our approach, we performed an evaluation on five benchmark color texture databases: Outex, NewBarktex2, USPTex, STex, and Parquet:

● Outex contains a very large number of surface textures acquired under controlled conditions by a 3-CCD digital color camera and whose size is 746×538 pixels. These textures are split up into 29 categories of color texture images such as wood, fabric, wallpaper, sand, tile,... [54]. To build the Outex set, 68 color texture images from 12 categories of this database are split up into 20 disjoint sub-images whose size is 128×128 pixels (see Figure 5 for a sample of each category), giving rise to 68 different texture classes. Among these 1360 sub-images, 680 are used for the training subset and the remaining 680 are considered as testing images. This dataset is known as the *Outex_TC_00013* test suite.
● The BarkTex database includes six tree bark classes, with 68 images per class. To build the NewBarkTex2 dataset, a region of interest, centered on the bark and whose size is 128×128 pixels, is first defined [2]. We thus obtain a set of 68 sub-images per class (see Figure 6 for a sample of each class). Half of these images are used for the training and the remaining 204 for the testing stage. Since the sub-images of this dataset come from different original images, the textures of the training and testing subsets are weakly correlated. This decomposition is available at: https://www-lisic.univ-littoral.fr/~porebski/NewBarkTex2.zip (accessed on 20 May 2022).
● USPtex contains 191 natural color textures acquired under an unknown but fixed light source [4]. The images are split up into 128×128 disjoint sub-images (see Figure 7 for randomly selected samples of different categories due to the large number of classes). Since the original image size is 512×384 pixels, this makes a total of 12 sub-images by a texture. For our experiments, this initial image dataset is split up in order to build a training and a testing image subset: six images are considered for the training and the six others are used as testing images. This decomposition is available at: https://www-lisic.univ-littoral.fr/~porebski/USPtex.zip (accessed on 27 January 2016).
● The Salzburg texture image database (Stex) is a large collection of 476 color texture images, whose acquisition conditions are not defined. Each of the 476 original images were split up into 16 non-overlapping 128×128 sub-images (see Figure 8 for randomly selected samples of different categories due to the large number of classes). For our experiments, this initial image dataset is split up in order to build a training and a testing image subset: eight images are considered for the training and the other eight are used as testing images. This decomposition is available at: https://www-lisic.univ-littoral.fr/~porebski/Stex.zip (accessed on 20 November 2017).
● The Parquet database is composed of fourteen varieties of wood for flooring [3]. Each type of wood presents several grades ranging from 2 to 4 which are considered as independent classes, leading to a total of 38 different classes. The main challenge with this database is that, within each type of wood, the grades are very similar to each other. Moreover, the sizes of the acquired images are different and the number of samples per class varies from 6 to 8. As in [13], six samples per class are retained and the images are center-cropped so that the final dimension of the images ranges from 480×480 to 1300×1300 pixels (see Figure 9 for a sample of different classes belonging to the OAK wood category). For each texture, three images are considered for the training and the other 3 are used as testing images. This decomposition is available at: https://www-lisic.univ-littoral.fr/~porebski/Parquet.zip (accessed on 10 June 2020).

Table 2 summarizes the color texture datasets used in the experiments of this paper.

### 5.2. Color Texture Feature Combinations

In order to highlight the contribution of combining several descriptor configurations, we propose to compare the CHMCS descriptor with different color texture representations, each coming from one descriptor:

- Single parameter setting and single color space (SPSC): this representation uses only one color space and a predefined parameter setting of the descriptor;
- Single parameter setting and multiple color space (SPMC): this representation uses multiple color spaces associated with a predefined parameter setting of the descriptor;
- Multiple parameter setting and single color space (MPSC): this representation uses only one color space and is based on several parameter settings of a same descriptor;
- Multiple parameter setting and multiple color space (MPMC): this representation uses multiple color spaces associated with several parameter settings of a same descriptor.

These various representations aim to determine which parameters really impact the classification results. To show this impact without being influenced by other parameters such as those of the classifier, we decide to use the nearest neighbor classifier because it does not need any parameter to adjust. As for the learning stage, this classifier is associated with the L1 distance and applied on the test image subsets. The accuracy is measured as the percentage of well-classified test images. All experiments were performed with the Matlab software using the CALCULCO computing platform supported by SCoSI/ULCO (Service Commun du Système d’Information from the University of Littoral Côte d’Opale) with different CPU and RAM. In addition, the online version of the CATAcOMB (Colour Furthermore, Texture Analysis Toolbox for Matlab) toolbox available at https://bitbucket.org/biancovic/catacomb accessed on 23 May 2022 and first released in February 2019 is used for comparisons with other approaches [13].

Table 3 indicates the number of features used for each of the color texture representations above presented and applied to different descriptors.

When these representations are associated with the dimensionality reduction scheme presented in the previous section, they give rise to other compact color texture descriptors that we compare in the next subsections. During the learning stage of these experiments, $d_{max}$ is set to 100 so that the learning processing time is reduced and *K* is set to 3 for the *K*-fold cross-validation which is repeated 10 times.

### 5.3. Extensive Analysis on the USPtex Dataset

First, we provide a detailed analysis of the results reached by the proposed approach on the USPtex dataset.

Table 4 shows the results obtained for each representation presented in the previous section with several color spaces and the parameter settings of different descriptors taken into account either individually or jointly. This table is organized as follows:

- For a given color space and a given parameter setting of a descriptor (RSCCM or EOCLBP), the accuracy obtained with the corresponding compact SPSC representation is indicated with the feature space dimension between brackets.
- For each parameter setting of a descriptor (RSCCM or EOCLBP) associated with multiple color spaces, the accuracy obtained with the corresponding compact SPMC representation is indicated in the last column with the feature space dimension between brackets. The previous two columns show, respectively, the mean accuracy and the accuracy interval computed from the five color spaces.
- For each color space associated with multiple parameter settings of a descriptor (RSCCM or EOCLBP), the accuracy obtained with the corresponding compact MPSC representation is indicated in the last row of the corresponding descriptor with the feature space dimension between brackets. The previous two rows show, respectively, the mean accuracy and the accuracy interval computed from the different parameter settings of the descriptor.
- For each descriptor, the accuracy obtained with the corresponding compact MPMC appears in the last corresponding cell (boxed value in the last column and last row).
- The three last rows give the results when the two descriptors (Hybrid: RSCCM and EOCLBP) are combined where the last cell indicates the accuracy obtained with the CHMCS descriptor (bold and boxed value).

The analysis of this table allows first to draw conclusions on the impact of SPMC (multiple color spaces) or MPSC (several parameter settings) representations compared to SPSC ones for each descriptor:

- By comparing the accuracy in the last column (SPMC) for each row (chosen parameter setting) of each descriptor with the maximum accuracy obtained with a single color space, the SPMC representation always outperforms the SPSC ones (+9.36% on average for the two descriptors) with higher dimensionality feature spaces (63.9 for SPMC and 30.9 for SPSC on average for the two descriptors). Thus, combining multiple color spaces improves the classification results compared to a single color space which is previously unknown.
- By comparing the accuracy in the last row (MPSC) for each column (chosen color space) of each descriptor with the maximum accuracy obtained with a single parameter setting, there are only two cases (out of 190) where the SPSC representation outperforms the SPMC ones. These two cases only appear with the parameter setting (256,1) of the RSCCM descriptor underlined in the table. On average, for the two descriptors, an accuracy increase of +10.18% is observed with the MPSC representation for a slight increase in the feature space dimensionality (42.0 for MPSC and 30.9 for SPSC). Compared to a single parameter setting which is previously unknown, the combination of several parameter settings globally improves the classification results.

For each descriptor independently considered, the compact MPMC (95.63% for RSCCM and 95.64% for EOCLBP) representation also provides higher accuracy than the best SPSC ones that is underlined and written in bold in Table 4 (94.24% for the (256,1) parameter setting of RSCCM in the YCbCr color space and 92.32% for the (12,2) parameter setting of the EOCLBP in the I1I2I3 color space). On average, MPMC representations provide higher accuracy than the SPMC (93.02% for RSCCM and 93.12% for EOCLBP) and MPSC (90.17% for RSCCM and 91.39% for EOCLBP) ones. However, there are three cases (underlined and written in italic in Table 4) where the SPMC representation, only with EOCLBP, outperforms a bit for this dataset. The combination of several descriptor configurations (multiple color spaces and parameter settings) is thus always preferred compared to representations where a predefined configuration is previously chosen (color space, parameter settings of the descriptor or both).

**Figure 10:**
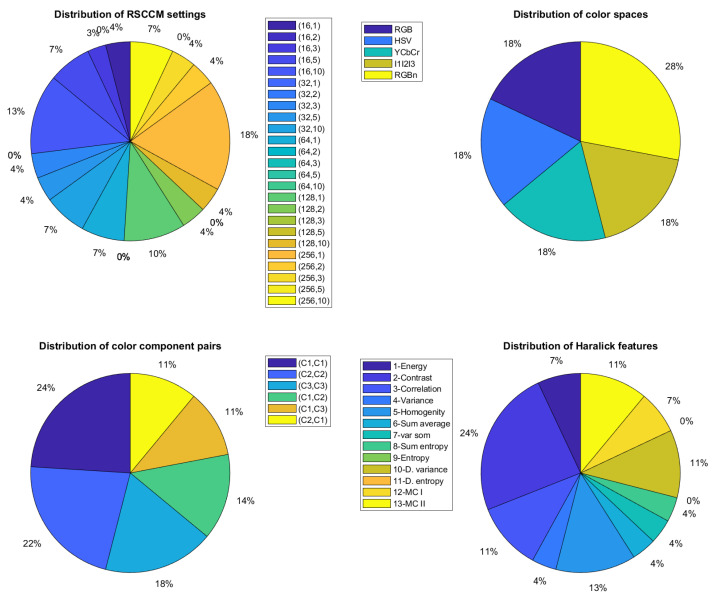
Distribution of the selected features for the RSCCM descriptor with the MPMC representation for the USPtex dataset. This distribution is shown for the descriptor settings, color spaces, color component pairs and Haralick features.

**Figure 11:**
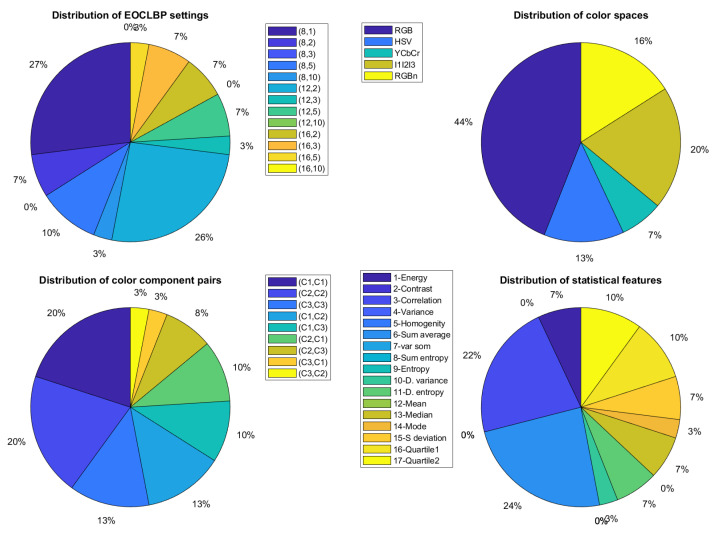
Distribution of the selected features for the EOCLBP descriptor with the MPMC representation for the USPtex dataset. This distribution is shown for the descriptor settings, color spaces, color component pairs and statistical features.

For each of the two descriptors used in this paper, Figure 10 and Figure 11 show, respectively, the distribution of the selected features when a MPMC representation is used. The distribution of color spaces, color component pairs, descriptor parameters, and features extracted from the descriptor are represented by pie charts.

For the RSCCM descriptor (see Figure 10), $\hat{d}=84$ color texture features are selected from the 9750 available ones with the USPtex dataset. These features are equally derived from the five spaces with the $R_{n}G_{n}B_{n}$ color space as the most selected (29%). The six possible pairs of components are also selected equally but the three within-component pairs represent more than half of the pairs. Among the 25 available combinations of parameters, 15 are exploited by the descriptor with quantization levels and neighborhood distances which are very different. Among 13 Haralick features, 11 are selected with a dominance of the contrast feature (25%).

For the EOCLBP descriptor (see Figure 11), $\hat{d}=89$ color texture features are selected from the 9945 available ones with the USPtex dataset. These features are derived from all color spaces with the RGB color space as the most selected (44%). All possible pairs of components are selected where the three within-component pairs represent more than half of the pairs. Among the 13 available combinations of parameters, 10 are exploited by the descriptor with different numbers of neighbors and neighborhood distances. The standard 3×3 isotropic 8-neighborhood seems to be the most often selected (27%). Among the 17 available statistical features, 10 are selected with a dominance of the homogeneity measure (24%).

This study confirms that, for a given descriptor, there is no unique color space, pair of components, parameter setting or feature extracted which is relevant. Indeed, the selected configuration varies with the considered descriptor. These results justify the approach proposed in this paper.

**Figure 12:**
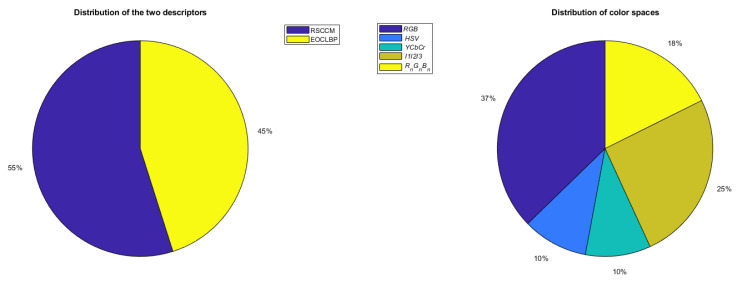
Distribution of the two descriptors and the five color spaces with the CHMCS representation for the USPtex dataset.

Finally, Table 4 shows that the proposed CHMCS descriptor (97.70% boxed and bold written in this table) outperforms any of all other representations. For the USPtex dataset, the two descriptors are relatively equally exploited and with all color spaces by the CHMCS representation (see Figure 12).

### 5.4. Overall Results

In this subsection, the rest of the results are given for the five color texture datasets.

For each of the two descriptors (RSCCM and EOCLBP), Table 5 first highlights the best results obtained with a compact SPSC representation. For each accuracy presented in this table, the corresponding descriptor configuration is given in terms of parameter setting and color space. This pair is different for the same descriptor on all datasets. This result allows to generalize the conclusion of the previous section.

Table 6 shows how the selected features of the CHMCS descriptor for each dataset are distributed. The second column of this table gives the dimension of the selected feature space which is lower than $d_{max}$ for all datasets. The next two columns count the number of times that each of the two descriptors are selected: in parentheses is the number of different parameter settings of this descriptor (25 settings are candidates for RSCCM and 13 settings are candidates for EOCLPB) and the number of its different color component pairs (6 pairs are candidates for RSCCM and 9 pairs are candidates for EOCLPB). For most datasets, this number is approximately equal except for the Parquet dataset where RSCCM is more often selected. For the NewBarkTex2 dataset, it can be pointed that it is EOCLBP which is selected a little more often. This table also shows that various parameter settings are used with the two descriptors. The six available component pairs are used by the RSCCM descriptor for four datasets (NewBarkTex2 only requires five pairs) while, for the EOCLPB, the number of used component pairs is between six and nine. The last five columns count the number of times each of the five color spaces is selected. All the color spaces are exploited by the CHMCS descriptor.

Table 7 gives the accuracy obtained on the five datasets with the proposed CHMCS descriptor compared to those obtained with the compact SPSC, MPSC, SPMC, and MPMC representations for each of the two descriptors. For the SPSC, SPMC and MPSC, the mean of the classification rates are given as the measure of accuracy. In order to underline the impact of the dimensionality reduction scheme, the accuracy obtained without selection is also given. For each dataset, the highest accuracy is written in bold and the highest accuracy reached by the other compact representations is underlined.

For all datasets, the CHMCS descriptor outperforms the other approaches and shows the purpose of combining different descriptors. When a single descriptor is considered (RSCCM or EOCLBP), compact representations that take into account several descriptor configurations (MPMC) give the highest accuracy. Compared to Table 5, wherein the best results appear with a predefined descriptor parameter setting and color space, the CHMCS descriptor provides a higher accuracy and solves the difficult problem of a prior choice of well-suited configuration. This is an essential key point of our approach. Classification results drastically decrease when no selection is performed. The dimensionality reduction scheme is thus the second essential key point for the success of the classification.

Table 8 indicates the dimensionality of the selected feature space from which the classification rates of Table 7 are computed. For the SPSC, SPMC and MPSC, the mean dimensionalities are computed. In all cases, the dimensions are less than $d_{max}=100$, which considerably reduces the classification time during the decision stage.

Finally, we propose to compare the relevance of the proposed CHMCS descriptor with handcrafted color texture descriptors and deep learning approaches (see Table 9). Deep learning, and more specifically, convolutional neural networks (CNNs) provide impressive performances in computer vision problems such as image classification, object detection or pattern recognition, and have become the benchmark computer vision technique of our time. For a fair comparison, the accuracy is thus evaluated on the five color texture datasets with image classification algorithms based on:

- Four popular pretrained CNN models that are fine-tuned with the training images of the considered dataset: AlexNet, GoogleNet, ResNet18 and ResNet50 [25,26,27]. Here, the last fully connected layer is modified to match the number of classes in each target dataset;
- Five pretrained generic CNN models that provided the best overall results in [13]: ResNet-50, ResNet-101, ResNet-152, VGG-VD-16 and VGG-VD-19 [27,28]. Here, the order-sensitive output of the last fully connected layer is used to generate the features and an L2 normalization of the resulting feature vector is achieved. The dimensionality of this vector is 2048 for the ResNet models and 4096 for the VGG-VD ones;
- Four handcrafted color texture descriptors: OCLBP, IOCLBP, LCVBP, and SWOBP (see Section 2) in addition to the best configuration determined for RSCCM and EOCLBP (see Table 5). OCLBP, IOCLBP, and LCVBP descriptors were used to obtain a multiple resolution feature vector by concatenating the histograms of their rotation-invariant version computed with five 8-neighborhood distances ($\delta \in \left\{1,2,3,5,10\right\}$). The dimensionality of the OCLBP, IOCLBP and LCVBP descriptors are thus $\left(36\times 5\times 6\right)=1080$, $\left(71\times 5\times 3+72\times 5\times 3\right)=2145$ and $\left(36\times 5\times 4\right)=720$, respectively. The SWOBP descriptor is used with the setting recommended by the authors (24 neighbors at a distance δ=3) so that its dimensionality is 2244 when the uniform pattern version is used.

Except for the fine-tuned CNN models where the classification is performed directly by the network, all the results are determined using the nearest neighbor classifier associated with the L1 norm distance.

This table shows that our approach based on supervised learning provides results which are consistent with those achieved with deep learning networks and superior to traditional handcrafted color texture descriptors. On average, it gives better results than pretrained CNN-based approaches. For two datasets (Outex and Parquet), the proposed texture representation outperforms the CNN-based representation with the highest accuracy (ResNet50) and provides the same result for the NewBarkTex2 dataset. The smaller the database is, the more the proposed descriptor seems to outperform this network. CNN-based methods fail when the number of training samples is low, as with the Parquet dataset, while our approach gives satisfactory results. Moreover, the CHMCS representation is able to discriminate the color textures of different classes which are very similar to each other as in the Parquet dataset, whereas the pretrained CNNs do not seem suitable for fine-grained texture classification with small inter-class and large intra-class variations, as shown in [13]. Thus, our approach remains very stable with any of the color texture dataset compared to CNNs. The results achieved by the proposed CHMCS representation with only two basic descriptors (RSCCM and EOCLBP) are very encouraging and lead to consider that they can be improved, even for large datasets, by adding other descriptors which provided notable results in Table 9.

## 6. Conclusions

In this paper, we proposed a compact, hybrid and multi-color space texture representation based on two key points:

- The combination of texture features extracted from several parameter settings of different descriptors computed from images coded in multiple color spaces.
  This representation simultaneously takes into account the different color and spatial properties of the textures to be analyzed and overcomes the difficulty of a prior parameter setting.
- The dimensionality reduction of the feature space by a clustering-based sequential forward selection procedure.
  The proposed selection procedure uses a feature subset search algorithm associated with a multi-criteria evaluation and is applied to the features automatically grouped into clusters beforehand. Feature clustering is performed from a dependency graph which is constructed using a *correlation measure*. Based on a *distance measure*, only one feature per cluster is selected at each iteration step of the sequential forward procedure and the cluster to which this feature belongs is removed from the feature set. Following an embedded model, the dimension of the feature space is finally determined by an *accuracy measure* computed from a validation image subset based on a repeated *K*-fold cross-validation.

In most traditional approaches, the texture descriptors are used with a predefined setting of the parameters and computed from images coded in a chosen color space. The principal contribution of the proposed approach is to combine the manifold configurations of descriptors including the color spaces in order to take into account the possible low inter-class and high intra-class appearance variations of the color textures to be classified. Another key point of this approach is the use of a correlation coefficient whose threshold is automatically determined by evaluating the feature clustering quality with a cluster separability and compactness measure so that no parameter requires adjustment

The results obtained with five benchmark texture databases show that combining the different configurations of a texture descriptor always improves the accuracy compared to approaches that use a prior predefined configuration. The proposed compact hybrid multi color space descriptor provides, on average, better results compared to deep learning approaches. Although the results obtained with the ResNet50 network are better when the number of data is large, this CNN-based representation fails when the dataset is small.

The proposed method could be extended by adding other descriptors in order to produce a better performance, even if the datasets are large. It can also be improved by adding criteria guaranteeing the stability of the feature selection procedure when the number of features is high compared to the number of training samples. Classification results could obviously be enhanced by using more sophisticated classifiers. However, our approach provides a high level of dimensionality reduction and competitive classification accuracy with a reasonable processing time and very few parameters to adjust.

## Figures and Tables

**Table 1:** Dimensionality of color texture feature spaces.

Descriptor			$N_{\mathrm{conf}}^{p}=$		Dimensionality
	p	$N_{\mathrm{spac}}$	$N_{\mathrm{pair}}^{p}\times N_{\mathrm{para}}^{p}$	$N_{\mathrm{feat}}^{p}$	
RSCCM	1	1	6×1	13	78
EOCLBP	2	1	9×1	17	153
RSCCM	1	5	6×1	13	390
EOCLBP	2	5	9×1	17	765
RSCCM	1	1	6×25	13	1950
EOCLBP	2	1	9×13	17	1989
RSCCM	1	5	6×25	13	9750
EOCLBP	2	5	9×13	17	9945

**Table 2:** Experimented color texture datasets.

Dataset	Image	Number of	Number of	Number of
	Size	Classes	Images	Images/Class
Outex	128×128	68	1360	20
NewBarkTex2	128×128	6	408	68
USPtex	128×128	191	2292	12
Stex	128×128	476	7616	16
Parquet	480×480 to	38	228	6
	1300×1300			

**Table 3:** Color texture representations where $N_{spac}$ is the number of considered color spaces, $N_{pair}^{p}$ is the number of color component pairs considered in each color space, $N_{para}^{p}$ is the number of parameter combinations, $N_{feat}^{p}$ is the number of color texture features extracted from each configuration of the pth descriptor, and *D* is the total number of color texture features.

Representation	Descriptor	*p*	$N_{\mathrm{spac}}$	$N_{\mathrm{pair}}^{p}$	$N_{\mathrm{para}}^{p}$	$N_{\mathrm{feat}}^{p}$	*D*
SPSC	RSCCM	1	1	6	1	13	78
SPSC	EOCLBP	2	1	9	1	17	153
SPMC	RSCCM	1	5	6	1	13	390
SPMC	EOCLBP	2	5	9	1	17	765
MPSC	RSCCM	1	1	6	25	13	1950
MPSC	EOCLBP	2	1	9	13	17	1989
MPMC	RSCCM	1	5	6	25	13	9750
MPMC	EOCLBP	2	5	9	13	17	9945
CHMCS	RSCCM + EOCLBP	–	5	–	–	–	19,695

**Table 4:** Classification accuracy (in % of well-classified test images) for the USPtex dataset. For each descriptor, the best result reached by an SPSC representation is written in bold and underlined. The results obtained with MPMC and CHMCS representations are boxed (and bold written for CHMCS). Other italic and/or underlined annotations refer to other discussed results.

Descriptor	Setting	RGB	HSV	YCbCr	I1I2I3	$R_{n}G_{n}B_{n}$	Min–Max	Mean	SPMC
**RSCCM**	(16,1)	82.98 (22)	91.27 (47)	88.83 (34)	89.35 (31)	68.76 (29)	[68.76–91.27]	84.24 (32.6)	93.11 (78)
	(16,2)	82.02 (27)	88.22 (32)	88.05 (30)	88.39 (24)	69.46 (30)	[69.46–88.39]	83.23 (28.6)	94.15 (69)
	(16,3)	80.11 (24)	89.44 (41)	85.95 (34)	86.56 (43)	66.41 (31)	[66.41–89.44]	81.69 (34.6)	93.19 (84)
	(16,5)	75.83 (13)	87.44 (41)	82.64 (38)	82.81 (29)	61.52 (30)	[61.52–87.44]	78.05 (30.2)	91.10 (70)
	(16,10)	71.99 (17)	82.98 (37)	76.00 (30)	77.75 (32)	58.55 (27)	[58.55–82.98]	73.46 (28.6)	88.22 (100)
	(32,1)	82.29 (23)	90.75 (39)	91.89 (41)	91.36 (36)	78.45 (28)	[78.45–91.89]	86.95 (33.4)	95.03 (99)
	(32,2)	82.98 (26)	89.97 (49)	90.40 (38)	88.74 (30)	75.65 (27)	[75.65–90.40]	85.55 (34.0)	95.03 (99)
	(32,3)	82.02 (23)	89.44 (46)	89.18 (32)	89.27 (30)	73.56 (28)	[73.56–89.44]	84.69 (31.8)	93.98 (98)
	(32,5)	78.01 (15)	87.87 (44)	85.69 (36)	86.48 (26)	71.38 (20)	[71.38–87.87]	81.88 (28.2)	91.01 (60)
	(32,10)	72.60 (15)	84.64 (34)	81.76 (32)	82.90 (31)	67.19 (23)	[67.19–84.64]	77.82 (27.0)	89.88 (88)
	(64,1)	83.51 (31)	90.93 (47)	92.76 (31)	93.11 (33)	82.37 (26)	[82.37–93.11]	88.53 (33.6)	94.85 (96)
	(64,2)	83.07 (29)	91.10 (41)	92.67 (40)	91.89 (27)	80.72 (23)	[80.72–92.67]	87.89 (32.0)	94.50 (96)
	(64,3)	82.02 (26)	89.79 (42)	91.27 (22)	90.75 (27)	77.92 (20)	[77.92–91.27]	86.35 (27.4)	93.19 (96)
	(64,5)	77.92 (21)	87.78 (42)	87.26 (25)	87.35 (22)	77.05 (17)	[77.05–87.78]	83.47 (25.4)	92.50 (94)
	(64,10)	72.51 (18)	84.03 (36)	82.72 (24)	84.56 (31)	72.86 (17)	[72.51–84.56]	79.34 (25.2)	89.79 (56)
	(128,1)	83.42 (33)	91.01 (37)	93.63 (34)	93.28 (32)	82.81 (27)	[82.81–93.63]	88.83 (32.6)	95.29 (88)
	(128,2)	82.90 (31)	90.66 (45)	91.97 (34)	92.76 (37)	80.98 (27)	[80.98–92.76]	87.85 (34.8)	94.24 (91)
	(128,3)	81.41 (28)	90.23 (39)	91.54 (28)	90.66 (37)	78.36 (19)	[78.36–91.54]	86.44 (30.2)	93.37 (78)
	(128,5)	78.01 (23)	88.31 (33)	88.83 (26)	88.83 (26)	74.87 (18)	[74.87–88.83]	83.77 (25.2)	93.11 (82)
	(128,10)	74.78 (19)	85.17 (38)	84.90 (23)	86.48 (30)	70.33 (18)	[70.33–86.48]	80.33 (25.6)	90.49 (56)
	(256,1)	83.33 (27)	91.89 (44)	94.24 (36)	93.37 (39)	85.17 (26)	[83.33–94.24]	89.60 (34.4)	94.94 (93)
	(256,2)	82.81 (32)	90.49 (45)	93.28 (35)	92.41 (37)	84.56 (27)	[82.81–93.28]	88.71 (35.2)	94.33 (95)
	(256,3)	82.46 (29)	89.35 (48)	92.41 (32)	90.31 (37)	83.07 (24)	[82.46–92.41]	87.52 (34.0)	94.59 (65)
	(256,5)	79.06 (24)	86.82 (34)	89.79 (27)	89.53 (31)	80.28 (20)	[79.06–89.79]	85.10 (27.2)	93.54 (56)
	(256,10)	74.08 (24)	84.12 (36)	86.39 (24)	86.13 (32)	75.65 (17)	[74.08–86.39]	81.27 (26.6)	92.15 (66)
	**Min–Max**	[71.99–83.51]	[82.98–91.89]	[76.00–94.24]	[77.75–93.37]	[58.55–85.17]	[58.55–94.24 ]	[73.46–89.60]	[88.22–95.29]
	**Mean**	79.69 (24.0)	88.55 (40.7)	88.56 (31.4)	88.60 (31.6)	75.12 (24.0)	[75.12–88.60]	84.10 (30.3)	93.02 (82.1)
	**MPSC**	84.21 (25)	91.62 (45)	93.54 (26)	93.46 (46)	88.05 (41)	[84.21–93.54]	90.17 (36.6)	95.63(84)
**EOCLBP**	(8,1)	83.68 (39)	87.20 (24)	89.79 (22)	88.37 (36)	80.19 (19)	[80.19–89.79]	85.85 (28.0)	92.90 (37)
	(8,2)	82.55 (28)	87.52 (40)	90.05 (27)	88.31 (26)	82.37 (23)	[82.37–90.05]	86.16 (28.8)	95.40 (41)
	(8,3)	84.47 (37)	86.13 (24)	91.10 (24)	89.41 (24)	83.71 (23)	[83.71–91.10]	86.96 (26.4)	94.59 (50)
	(8,5)	77.57 (44)	84.47 (33)	84.53 (21)	84.03 (38)	79.32 (24)	[77.57–84.53]	81.98 (32.0)	91.45 (40)
	(8,10)	60.30 (46)	71.64 (50)	72.51 (50)	73.56 (49)	71.73 (27)	[60.30–73.56]	69.95 (44.4)	87.26 (46)
	(12,2)	84.99 (26)	87.87 (27)	90.31 (16)	92.32 (33)	83.33 (24)	[83.33−92.32]	87.77 (25.2)	96.28 (50)
	(12,3)	84.73 (34)	88.31 (19)	90.84 (36)	90.05 (32)	84.99 (23)	[84.73–90.84]	87.78 (28.8)	95.38 (37)
	(12,5)	82.72 (43)	85.34 (47)	86.56 (39)	88.37 (29)	84.21 (20)	[82.72–88.37]	85.44 (35.6)	93.28 (48)
	(12,10)	65.10 (26)	76.06 (50)	75.92 (45)	73.33 (27)	70.94 (34)	[65.10–76.06]	72.27 (36.4)	86.13 (47)
	(16,2)	85.08 (28)	89.35 (25)	91.80 (32)	91.45 (34)	83.45 (33)	[83.45–91.80]	88.23 (30.4)	96.16 (50)
	(16,3)	87.00 (27)	89.09 (31)	92.06 (23)	91.45 (30)	84.50 (18)	[84.50–92.06]	88.82 (25.8)	96.86 (49)
	(16,5)	82.37 (44)	85.69 (34)	88.57 (26)	89.79 (36)	84.03 (23)	[82.37–89.79]	86.09 (32.6)	93.89 (50)
	(16,10)	68.76 (32)	78.01 (36)	79.67 (31)	76.88 (31)	76.18 (48)	[68.76–79.67]	75.90 (35.6)	91.01 (50)
	**Min–Max**	[60.30–87.00]	[71.64–89.35]	[72.51–92.06]	[73.33–92.32]	[70.94–84.99]	[60.30–92.32]	[69.95–88.82]	[86.13–96.86]
	**Mean**	79.18 (34.9)	84.36 (33.9)	86.44 (30.1)	85.95 (32.7)	80.69 (26.1)	[79.18–86.44]	83.32 (31.5)	93.12 (45.8)
	**MPSC**	89.53 (50)	91.27 (29)	94.59 (42)	94.59 (37)	87.00 (29)	[87.00–94.59]	91.39 (37.4)	95.64(89)
**Hybrid**	**Min–Max**	[60.30–87.00]	[71.64–91.89]	[72.51–94.24]	[73.33–93.37]	[58.55–85.17]	[58.55–94.24]	[69.95–89.60]	[86.13–96.86]
	**Mean**	79.43 (29.45)	86.45 (37.2)	87.50 (30.7)	87.27 (32.1)	77.90 (25.0)	[77.90–87.50]	83.71 (30.9)	93.07 (63.9)
	**Hybrid**	94.68 (49)	94.85 (31)	95.55 (49)	96.68 (51)	87.69 (30)	[87.69–96.68]	93.89 (42.0)	**97.70(51)**

**Table 5:** Accuracy (in % of well-classified test images) obtained by the best configuration of each descriptor.

Dataset	RSCCM			EOCLBP		
	Best Result	Setting	Color Space	Best Result	Setting	Color Space
Outex	94.41	(128,2)	RGB	91.91	(12,3)	HSV
NewBarkTex2	80.39	(32,5)	YCbCr	82.37	(8,2)	YCbCr
USPtex	94.24	(256,1)	YCbCr	92.32	(12,2)	I1I2I3
Stex	89.57	(64,1)	I1I2I3	90.04	(16,2)	HSV
Parquet	85.96	(16,1)	HSV	78.94	(8,1)	HSV

**Table 6:** Distribution of the selected features.

Dataset	$\hat{d}$	Descriptor				Color Spaces				
		RSCCM (25, 6)		EOCLBP (13, 9)		RGB	HSV	YCbCr	I1I2I3	$R_{n}G_{n}B_{n}$
Outex	36	17	(9, 6)	19	(9, 8)	12	8	6	5	5
NewBarkTex2	20	7	(6, 5)	13	(8, 7)	3	3	2	6	6
USPtex	51	28	(15, 6)	23	(11, 9)	19	5	5	13	9
Stex	75	37	(18, 6)	38	(10, 6)	19	20	16	9	11
Parquet	41	29	(12, 6)	12	(8, 9)	12	5	10	5	9

**Table 7:** Accuracy obtained (in % of well-classified test images) with the CHMCS descriptor compared to other approaches. For each dataset, the highest accuracy is written in bold. The highest accuracy reached by the other approaches is underlined. “With” and “Without” refer to the selection procedure.

Dataset	RSCCM				EOCLBP				CHMCS	
	SPSC	SPMC	MPSC	MPMC	SPSC	SPMC	MPSC	MPMC	Without	With
Outex	89.89	93.84	92.70	95.44	84.32	91.43	87.88	92.35	91.76	95.59
NewBarkTex2	69.97	77.65	71.76	82.84	74.39	83.82	88.82	90.69	87.25	94.61
USPtex	84.10	93.02	90.17	95.63	83.32	93.12	91.39	95.64	54.89	97.70
Stex	78.62	89.72	85.58	92.62	79.28	90.53	89.29	94.04	70.25	96.06
Parquet	74.66	80.21	79.29	83.33	69.33	71.93	69.82	73.68	57.89	86.84

**Table 8:** Dimensionality of the feature space obtained with the CHMCS descriptor compared to other approaches for each dataset.

Dataset	RSCCM				EOCLBP				CHMCS
	SPSC	SPMC	MPSC	MPMC	SPSC	SPMC	MPSC	MPMC	
Outex	11.44	17.28	13.00	27	26.43	33.00	31.00	59	36
NewBarkTex2	15.10	29.56	9.00	24	18.34	24.23	26.80	27	20
USPtex	30.33	82.12	36.60	84	31.53	45.74	37.40	89	51
Stex	29.63	29.60	87.04	83	36.38	47.15	39.40	67	75
Parquet	5.76	12.88	12.60	15	8.00	7.38	11.20	8	41

**Table 9:** Comparison with handcrafted descriptors and deep learning approaches. Mean classification rates are provided (written in italic) for each approach across the five datasets. For each dataset, the best results are written in bold.

Descriptor	Dataset					Average
	Outex	NewBarkTex2	USPtex	Stex	Parquet	
*Our approach*						
CHMCS	95.59	94.61	97.70	96.06	86.84	94.16
*Fine-tuned CNN models*						
ResNet50	86.03	94.61	99.39	97.30	64.04	88.27
Resnet18	84.12	89.71	96.60	95.22	51.75	83.48
GoogleNet	80.29	88.73	97.12	92.70	42.98	80.36
AlexNet	86.03	91.18	93.02	91.44	50.00	82.33
*Pretrained generic CNN models*						
ResNet-50	89.26	91.18	99.48	97.27	54.39	86.32
ResNet-101	88.09	90.69	98.95	96.48	56.14	86.07
ResNet-152	85.74	89.71	99.04	96.82	58.77	86.02
VGG-VD-16	85.44	84.31	96.95	94.51	51.75	82.59
VGG-VD-19	85.15	87.25	97.29	94.62	47.37	82.34
*Traditional handcrafted color texture descriptors*						
IOCLBP	93.09	69.61	93.54	91.70	68.42	83.27
OCLBP	94.12	72.55	93.19	90.76	69.30	83.98
LCVBP	90.00	89.22	94.33	91.81	75.44	88.16
SWOBP	86.32	90.20	94.15	90.20	69.30	86.03
*Best RSCCM*	94.41	80.39	94.24	89.57	85.96	88.91
*Best EOCLBP*	91.91	82.37	92.32	90.04	78.94	87.12
